# De novo mutations in *FBRSL1* cause a novel recognizable malformation and intellectual disability syndrome

**DOI:** 10.1007/s00439-020-02175-x

**Published:** 2020-05-18

**Authors:** Roser Ufartes, Hanna Berger, Katharina Till, Gabriela Salinas, Marc Sturm, Janine Altmüller, Peter Nürnberg, Holger Thiele, Rudolf Funke, Neophytos Apeshiotis, Hendrik Langen, Bernd Wollnik, Annette Borchers, Silke Pauli

**Affiliations:** 1 Institute of Human Genetics, University Medical Center Göttingen, Heinrich-Düker-Weg 12, 37073 Göttingen, Germany; 2 Department of Biology, Molecular Embryology, Philipps-University Marburg, Marburg, Germany; 3 NGS Integrative Genomics Core Unit, University Medical Center Göttingen, 37073 Göttingen, Germany; 4 Institute of Medical Genetics and Applied Genomics, Calwerstr. 7, 72076 Tübingen, Germany; 5 Cologne Center for Genomics (CCG), University of Cologne, Weyertal 115b, 50931 Cologne, Germany; 6 Center for Molecular Medicine Cologne (CMMC), University of Cologne, Robert-Koch Str. 21, 50931 Cologne, Germany; 7 Department of Neuropediatrics, Sozialpädiatrisches Zentrum, Mönchebergstr. 41-43, 34125 Kassel, Germany; 8 Praxis für Humangenetik, Georg-Eckert-Straße 12, 38100 Brunswick, Germany; 9 Department of Neuropediatrics, Sozialpädiatrisches Zentrum Hannover, Janusz-Korczak-Allee 8, 30173 Hannover, Germany; 10 Cluster of Excellence “Multiscale Bioimaging: From Molecular Machines To Networks of Excitable Cells” (MBExC), University of Göttingen, 37073 Göttingen, Germany; 11 DFG Research Training Group, Membrane Plasticity in Tissue Development and Remodeling, GRK 2213, Philipps-University Marburg, Marburg, Germany

## Abstract

We report truncating de novo variants in specific exons of *FBRSL1* in three unrelated children with an overlapping syndromic phenotype with respiratory insufficiency, postnatal growth restriction, microcephaly, global developmental delay and other malformations. The function of *FBRSL1* is largely unknown. Interestingly, mutations in the *FBRSL1* paralogue *AUTS2* lead to an intellectual disability syndrome (AUTS2 syndrome). We determined human *FBRSL1* transcripts and describe protein-coding forms by Western blot analysis as well as the cellular localization by immunocytochemistry stainings. All detected mutations affect the two short N-terminal isoforms, which show a ubiquitous expression in fetal tissues. Next, we performed a Fbrsl1 knockdown in *Xenopus laevis* embryos to explore the role of Fbrsl1 during development and detected craniofacial abnormalities and a disturbance in neurite outgrowth. The aberrant phenotype in *Xenopus laevis* embryos could be rescued with a human N-terminal isoform, while the long isoform and the N-terminal isoform containing the mutation p.Gln163* isolated from a patient could not rescue the craniofacial defects caused by Fbrsl1 depletion. Based on these data, we propose that the disruption of the validated N-terminal isoforms of FBRSL1 at critical timepoints during embryogenesis leads to a hitherto undescribed complex neurodevelopmental syndrome.

**Electronic supplementary material:**

The online version of this article (10.1007/s00439-020-02175-x) contains supplementary material, which is available to authorized users.

## Introduction

In two unrelated children with a nearly identical clinical phenotype we performed trio-exome sequencing to uncover the underlying cause of a hitherto undiagnosed congenital malformation syndrome. In both affected children, we detected a de novo truncating variant in *FBRSL1.* Single exome sequencing and “reverse phenotyping” revealed a third patient having a truncating *FBRSL1* mutation. The function of FBRSL1 is largely unknown and so far this gene has not been associated with any syndromic phenotype. Interestingly, mutations in the *FBRSL1* paralogue *AUTS2* (NG_034133.1, *activator of transcription and developmental regulator*) were first described in 2013 as causative of an intellectual disability syndrome with microcephaly and, in some cases, additional features like heart defects and contractures (AUTS2 syndrome, OMIM 615834) (Beunders et al. 2013). Our patients presented with features of the AUTS2 syndrome and additional symptoms, suggesting that mutations in *FBRSL1* produce a more complex phenotype than mutations in the paralogue *AUTS2*.

AUTS2 and FBRSL1 are components of the Polycomb subcomplexes PRC1.3 and PRC1.5 (Gao et al. 2014; Scelfo et al. 2015; Chittock et al. 2017). Polycomb complexes fulfill an important function during embryonic development by inactivating numerous target genes (e.g., HOX genes and transcription factors) at specific developmental timepoints (Müller and Verrijzer 2009; Simon and Kingston 2013; Schuettengruber et al. 2017). Polycomb repressive complexes are divided into different subgroups according to their function and additional complex partners (Gao et al. 2012). While PRC1 complexes exert their repressive function by chromatin compaction and H2AK119 monoubiquitination via the E3 ligases RING1A or RING1B, PRC2 complexes act by catalyzing methylation of the repressive histone mark H3K27 (H3K27me3) (Simon and Kingston 2013).

Interestingly, binding of AUTS2 to the respective polycomb complex (PRC1.3 or PRC1.5) inhibits the repressive function; the AUTS2-PRC complex thus acts as a transcriptional activator. This is achieved by the recruitment of histone acetyltransferase p300, a known transcriptional co-activator, and casein kinase 2, which inhibits the repressive PRC1 function (Gao et al. 2014; Hori and Hoshino 2017). It is unknown whether an FBRSL1-PRC complex acts in a repressive or activating manner.

Mutations in members of the PRC1 and PRC2 complex as well as in trithorax group proteins, counterparts of polycomb complexes, underlie numerous malformation and intellectual disability syndromes often caused by de novo mutations (Ng et al. 2004, 2010; Vissers et al. 2004; Lederer et al. 2012; Awad et al. 2013; Deevy and Bracken 2019). Therefore, it is likely that further uncharacterized PRC-associated proteins, like FBRSL1, are also associated with a malformation syndrome.

Here, we report the clinical presentation and molecular etiology of a novel syndrome and the functional analysis of *FBRSL1*, the involved gene.

## Material and methods

### Variant identification (whole-exome sequencing)

Whole-exome sequencing was performed on a blood DNA sample of patient 3 using Agilent SureSelect Human All Exon V6 r2 kit on an Illumina HiSeq4000 sequencer. Trio exome sequencing was performed on blood DNA samples of patients 1 and 2 and their parents using Agilent's SureSelectXT Human All Exon V5 Enrichment method. Trio analysis of samples of patient 1 and 2 was performed using the megSAP pipeline developed at the medical genetics department in Tübingen (https://github.com/imgag/megSAP). The exome data of patient 3 were analyzed using the “Varbank” pipeline of the Cologne Center for Genomics (CCG) with following filter criteria: coverage of > 6 reads, quality score of > 10, allele frequency of ≥ 25%, and a minor allele frequency (MAF) of < 0.1% in the 1000 Genomes database and the Exome Variant Server (EVS; NHLBI Exome Sequencing Project). In patient 3, the results were analyzed by the multidisciplinary NGS data analysis team [Mutation Mining (MM)-Team] of the Institute of Human Genetics, University Medical Center Göttingen.

### Transcript identification/RNA studies

To investigate the expression of different transcripts, RNA from lymphocytes was isolated and two µg RNA was reverse transcribed using Random Primers (SuperScript, Invitrogen). The different transcripts were amplified by PCR using specific primer and the Q5^®^ High-Fidelity DNA Polymerase (NEB). Sanger sequencing was performed to confirm the correct sequence. Afterwards, three transcripts were analyzed in different organs using a human adult and fetal cDNA panel from Clontech.

Sequencing of RNA-seq samples was conducted at the NGS Integrative Genomics Core Unit, University Medical Center Göttingen. Sequence images were transformed with Illumina software BaseCaller to BCL files, which was demultiplexed to fastq files with bcl2fastq v2.17.1.14. The sequencing quality was asserted using FastQC (Andrews 2014). FastQC A Quality Control tool for High Throughput Sequence Data. (https://www.bioinformatics.babraham.ac.uk/projects/fastqc/) (version 0.11.5). Sequences were aligned to the reference genome Homo sapiens (hg38 version 89, https://www.ensembl.org/Homo_sapiens/Info/Index) using the STAR aligner (Dobin et al. 2013) (version 2.5.2a) allowing for 2 mismatches within 50 bases. Subsequently, read counting was performed using featureCounts (Liao et al. 2014) (version 1.5.0-p1). Reads mapped to the *FBRSL1* gene were visualized using Integrated Genome Viewer (IGV) version 2.8.2.

### Plasmid preparation

The full-length human wild-type *FBRSL1* isoform 1 (NCBI, NM_001142641.1) and 3.1 (NCBI, XM_005266181.4) in the vector pcDNA3.1 ( +)-N-HA were synthetized by GenScript. Isoform 3.2 (ENSEMBL, ENST00000542061.2,) was amplified by PCR using DNA from human lymphocytes. The PCR product was cloned into the pCMV-HA vector (Clontech) by using the In-Fusion® HD Cloning Kit (Clontech) according to the manufacturer's protocol. To introduce the nonsense mutation, c.487C > T (p.Gln163*), site-directed mutagenesis PCR was performed by using the QuikChange II XL Site-Directed Mutagenesis Kit (Agilent) according to the manufacturer's protocol.

Sanger sequencing was performed for each plasmid to confirm the correct sequence. Sequences of used primers can be sent on request.

### Western blot analysis

Total lysate was isolated from HEK293 cells using modified RIPA Buffer. 30 µg protein were separated by a 4–12% NuPAGE Bis–Tris Gel (Invitrogen) and transferred to a Protran nitrocellulose membrane (0.45 µm) (Sigma-Aldrich) using a Tank system (Bio-Rad) at 250 mA for 60 min at 4 °C. After blocking the membrane with 5% milk/TBST (Carl Roth) for 60 min, the membrane was incubated with appropriate primary antibodies overnight at 4 °C. Rat anti-HA (11867431001, Sigma-Aldrich) was used at a dilution of 1:2000 in 2% milk/TBST. Rabbit anti-FBRSL1 (HPA049880, Sigma-Aldrich) was used at a dilution of 1:500 in 2% milk/TBST. After washing, the membrane was incubated with the following secondary antibodies: anti-rat IgG peroxidase secondary antibody (Sigma-Aldrich) at a dilution of 1:10,000 in 2% milk/TBST, goat IgG anti-rabbit (H + L)-HRPO (Dianova) at a dilution of 1:10,000 in 2% milk/TBST. The membrane was washed and Immobilon Western Chemiluminescent HRP Substrate (Merck Millipore) was applied on the membrane. Bands were detected with the Azur detecting system.

### Cell culture and transient transfection

HEK293 cells were cultured in Dulbecco's modified Eagle's medium (DMEM) (PAN-Biotech), supplemented with 10% fetal bovine serum (FBS) (PAN-Biotech), 1 × MEM Non-Essential Amino Acids (NEAA) (Thermo Fisher Scientific) and 1% penicillin/streptomycin (Thermo Fisher Scientific). For transfection, 5 × 10^6^ HEK293 cells were plated into T75 culture flasks (Greiner Bio-One). 24 h after plating the cells, cells were transfected with 10-µg plasmid using jetPrime (Polyplus) according to the manufacturer's instructions. Medium was changed four hours after transfection. 24 h after transfection, cells were harvested for further analysis. For immunocytochemistry experiments, 2 × 10^5^ HEK293 cells were plated into a 12-well plate on 12 mm cover slips (YX03.1, Roth). Cover slips were coated with 0.1% poly-lysine (P2636, Sigma-Aldrich) 4 h before plating the cells. 24 h after plating, the cells were fixed with 4% paraformaldehyde (PFA) for 10 min at room temperature (RT).

### Immunocytochemistry

Fixed cells were permeabilized with 0.5% Triton X-100 in PBS for 10 min, washed twice with 0,1% Tween in PBS (PBT) and blocked with 3% BSA in PBT for 30 min at RT. Afterwards primary antibodies in blocking buffer (1:100 anti-FBRSL1-N-terminal, Sigma-Aldrich, 1:100 anti-FBRSL1-C-terminal, Sigma-Aldrich, 1:2000 mouse monoclonal anti-α-tubulin, Sigma-Aldrich) were used overnight at 4 °C. After incubation, cells were washed with PBT and incubated for 1 h at RT with secondary antibodies (1:600 goat anti-rabbit IgG (H + L)-Alexa Fluor 488, 1:600 goat anti-mouse IgG (H + L)-Alexa Fluor 546; Invitrogen). Subsequently, cells were washed twice and mounted using Fluoroshield™ with DAPI (Merck Millipore). Immunofluorescent images were acquired using the Olympus BX60 microscope with × 600 magnification, and an additional software magnification of 3, 6, 9 and 12. All images were processed with FIJI (an image-processing package based on ImageJ). All experiments were performed three times.

### Studies in *Xenopus laevis*

#### *Xenopus* microinjection

*Xenopus laevis* embryos were obtained and cultured using standard protocols and staged according to the normal table of Nieuwkoop and Faber (Nieuwkoop and Faber 1956). All procedures were performed according to the German Animal Use and Care Act (Tierschutzgesetz) and approved by the German state administration Hesse (Regierungspräsidium Giessen). For phenotypical analysis, *Xenopus* embryos were injected in one blastomere at the two-cell stage and for RT-PCR at the one-cell stage. Capped sense mRNA for microinjection was synthetized using the mMessage mMachine™ SP6 Transcription Kit (Invitrogen™) from the following plasmids: lacZ (Smith and Harland 1991) and mGFP (Moriyoshi et al. 1996). The following Morpholino Oligonucleotides (MO) were used for microinjections: Standard control morpholino (Co MO, 5′-CCTCTTACCTCAGTTACAATTTATA-3′, Gene Tools, LLC) and *fbrsl1* E1/I1 splice MO (*fbrsl1* MO, 5′- ATAACTCTCTCTTACCTCTAAGGCT-3′, Gene Tools, LLC). For rescue experiments plasmid microinjection using the human FBRSL1 transcript variants, FBRSL1 isoform 1, FBRSL1 isoform 3.1 and FBRSL1 isoform 3.1-p.Gln163* were performed.

#### Phenotypical characterization of *Xenopus laevis* embryos

For phenotypical characterization of the craniofacial structures, 100 pg *lacZ* mRNA were co-injected as lineage tracer. Embryos were fixed in MEMFA (3.7% formaldehyde, 0.1 M MOPS, 2 mM EGTA, 2 mM MgSO_4_) and the injected side was visualized by X-gal staining. For phenotypical documentation of craniofacial defects, a Leica M165 FC stereo microscope with a DFC450C Camera was used.

#### Whole-mount immunofluorescence staining of *Xenopus laevis* embryos

For whole-mount immunofluorescence staining, the embryos were co-injected with 50 pg *mGFP* mRNA as a lineage tracer. Embryos were fixed in Dent’s fixative (20% DMSO, 80% methanol), washed twice in PBS, and subsequently photobleached in 2% H_2_O_2_ in PBS under a light source until the embryos lost their pigmentation. After photobleaching, the embryos were washed twice in PBS-TD (1% Triton X-100, 1% DMSO, 1 × PBS) and blocked for two hours in blocking buffer (0.1 M glycine, 2% nonfat dried milk, 5% FBS in PBS-TD) at room temperature. Afterwards, the embryos were rinsed in PBS-TD and treated with 1 mg/ml bovine testicular hyaluronidase (Sigma Aldrich) in 50 mM sodium acetate buffer (pH 6) for 45 min at RT. The samples were washed in PBS-TD, blocked again for 30 min in blocking solution and incubated with the primary antibody anti-Collagen Type II (DSHB, II-II6B3) or anti-Ncam (DSHB, 4d) diluted 1:50 in blocking buffer overnight at 4 °C. The following day, six wash steps in PBS-TD of 30 min to 1 h each were performed following incubation with the secondary antibody Alexa Fluor^®^ 594 Goat anti-Mouse (Invitrogen™, A-11005, diluted 1:400 in blocking buffer) overnight at 4 °C. After overnight incubation, again six wash steps in PBS-TD of 30 min to 1 h each were performed and the embryos were subsequently re-fixed in Dent’s fixative overnight at 4 °C. Before imaging, embryos were washed twice in 100% ethanol and incubated in Benzyl-alcohol/Benzyl-benzoate (BA/BB, 1:2) in glass dishes for 10 min. Imaging of the stained cartilage was performed in fresh BA/BB in glass dishes using a Leica M165 FC stereo microscope with a DFC450C Camera. Brain and cartilage phenotypes were quantified by measuring the area of both brain hemispheres or the cartilage (ceratohyal cartilage and branchial arches), respectively, using the polygon function of ImageJ. The relative surface area between control side and Morpholino injected side was calculated by setting the control side to 100%. The embryos were classified as having defects, if the brain or the cartilage area of the injected side was reduced by at least 15% compared to the control side.

### Statistical analysis

To analyze the significance of the statistical data, a two-tailed unpaired Student’s *t*-test and a one-way ANOVA test were applied with the indicated *p *values: *p ≤ 0.05, **p ≤ 0.01, ***p ≤ 0.001.

### RT-PCR (*Xenopus laevis)*

To verify the *fbrsl1* splice-blocking effect of the *fbrsl1* Morpholino total RNA was isolated from stage 30 embryos injected with 10 ng Co MO or 10 ng *fbrsl1* MO. To analyze the temporal expression pattern of *fbrsl1* in *Xenopus* embryos, RNA was isolated from oocytes and embryos of different developmental stages. Five embryos per condition were used for RNA isolation using the GE Healthcare Illustra RNAspin Mini Isolation Kit according to the manufacturer’s instructions. 2 µg of the isolated RNA served as template for cDNA synthesis and was mixed with 0.2 µg Random Hexamer Primer (Thermo Scientific) in a volume of 13.5 µl and incubated at 65 °C at 5 min. After incubation, 1 mM dNTP Mix (Thermo Scientific), 20 units MuLV Reverse transcriptase (Thermo Scientific), 5 × Reaction Buffer (Thermo Scientific) and 20 units RNaseOUT (Invitrogen™) and bidest H_2_O were added to a final volume of 20 µl. The reaction was incubated for 10 min at 25 °C following incubation for 1 h at 42 °C. The following primers were used to verify the *fbrsl1* Morpholino: forward primer (5′-ATGGATATTAAAACCAAACAACCAAGCAGG-3′) and reverse primer (5′-ACAGAGGGTAAGGGGGAAGTT-3′). To analyze the temporal *fbrsl1* expression pattern the forward primer (5′-ATGGATATTAAAACCAAACAACCAAGCAGG-3′) and the reverse primer (5′-GTGAGACGTGGAGGAGCTGG-3′) were used to amplify a 942 bp *fbrsl1* fragment. As control histone H4 was amplified using the forward primer (5′-CGGGATAACATTCAGGGTATCACT-3′) and the reverse primer (5′-ATCCATGGCGGTAACTGTCTTCCT-3′). PCR products were separated on an agarose gel and detected using the Odyssey^®^ Fc Imaging System (LI-COR Bioscience).

## Results

### Identification of *FBRSL1* variants in three patients with a recognizable malformation syndrome

**Fig. 1:**
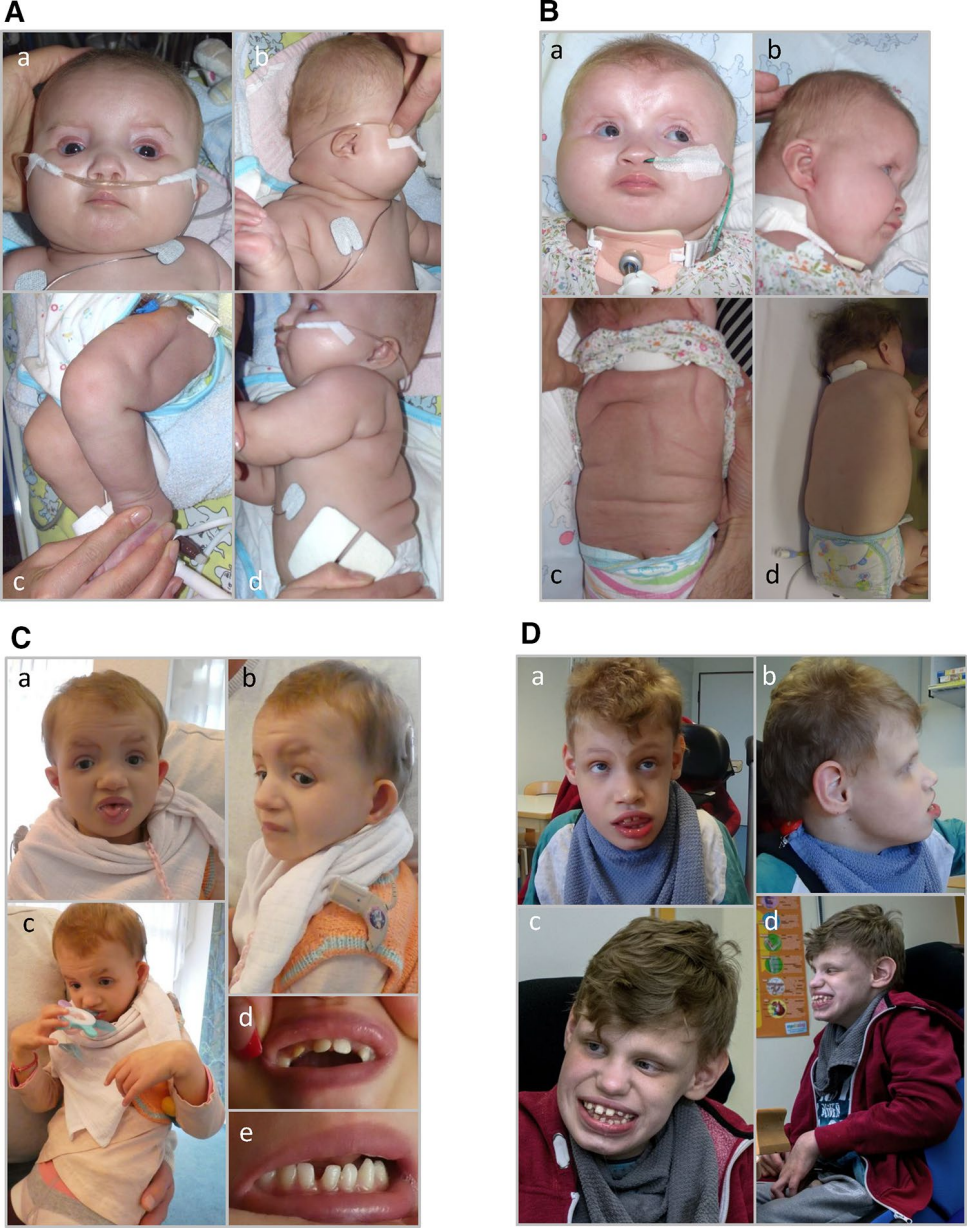
Clinical representation of the three patients. **A** Patient 1 at the age of six months: **a** part of the face **b** side view showing a pronounced neck fold, a flat back of the head and a dysmorphic auricle **c** side view of the right leg with wrinkles and **d** side view with a view of the back area with pronounced skin fold formation. **B** Patient 2 at the age of five months: **a** front view showing deep-set eyes, a round face and temporal indentations; **b** side view, which also shows a flat back of the head and a slightly dysplastic auricle. Due to the fixation of the tracheostoma, the pronounced neck fold is not visible; **c** view of the back with wrinkles; **d** view of the back of patient 2 at the age of two and a half years. A remarkable regression of the skin folds was observed. **C** Patient 1 at the age of 6 years and 7 months: **a** front-view and **b** side-view showing dysmorphic features. **c** Contractures on both hands and fingers are shown (**d**, **e**) view of the teeth. **D** front-view (**a**) and side-view (**b**) of patient 3 at the age of 12 years and 6 months. **c**, **d** facial appearance of patient 3 at the age of 14 years and 8 months. Contractures of both hands and fingers are observed, as well as wide-spaced teeth

Two unrelated children presented with respiratory insufficiency, feeding difficulties, postnatal growth restriction and microcephaly, global developmental delay, no active speech, contractures, heart defects, cleft palate, facial dysmorphism and distinctive skin creases in the first year of life. The skin phenotype was most pronounced immediately after birth and regressed in both children with increasing age. In contrast to the skin phenotype observed in children affected by the congenital symmetric circumferential skin creases syndrome (CSCSC1, OMIM 156,610; and CSCSC2, OMIM 616,734), the back was preferentially affected in our patients and not the arms and legs (Fig. 1). The clinical presentation could not be assigned to a known syndrome, although there are overlapping features to CSCSC1, CSCSC2 and AUTS2 syndrome (AUTS2 syndrome, OMIM 615,834).

We performed trio-exome sequencing to uncover the underlying cause. In both affected children, we excluded the presence of pathogenic variants in the genes *TUBB* and *MAPRE2* causing CSCSC1 and CSCSC2, respectively, and mutations in *AUTS2*. However, a de novo truncating variant in *FBRSL1*, an uncharacterized *AUTS2* paralogue, was identified in both children. The clinical findings in comparison to the published phenotype in AUTS2 syndrome patients are summarized in Table 1.

**Table 1:** Summary of the observed clinical findings in comparison to the published phenotype in AUTS2 syndrome patients

Clinical description (HPO term)	*FBRSL1* mutations			AUTS2 syndrome
	Patient 1 c.487C > T, p.Gln163*; de novo	Patient 2 c.581_603del; de novo	Patient 3 c.332 G > A; p.Trp111*; n.a	Beunders et al. (2015), (2016)
Intellectual disability (HP:0001249) and/or global development delay (HP:0001263)	+	+	+	+
Delayed speech and language development (HP:0000750)	+	+	+	+
Autistic behavior (HP:0000729)	+	+	+	+
Microcephaly (HP:0000252)	+	+	+	+
Swallowing difficulty (HP:0002015)	+	+	+	+
Postnatal growth retardation (HP:0008897)	+	+	+	+
Abnormality of the skeletal system (HP:0000924)	+	+	+	+
Camptodactyly/contractures (HP:0001371)	+	+	+	+
Heart defect (HP:0001627)	ASD/PDA	ASD/VSD	−	Atrial septum defect (ASD)
Cleft palate (HP:0000175)	+	+	−	−
Respiratory failure (HP:0002878) with ventilation therapy	+	+	+	−
Asplenia (HP:0001746)	−	+	−	−
Abnormality of the anus (HP:0004378)	+	−	−	−
hearing impairment (HP:0000365)	+	+	−	−
Abnormality of the skin (HP:0000951) skin creases	+	+	−	−
Facial dysmorphism (HP:0001999)	+	+	+	−

*n.a. *father not available for testing, *ASD *atrial septal defect, *VSD *ventricular septal defect, *PDA *persistent ductus arteriosus botalli, *HPO *human phenotype ontology

In patient 1 we identified in a heterozygous state the de novo mutation c.487C > T (12:133,085,942; GRCh37/hg19) in *FBRSL1*, leading to a premature stop codon (p.Gln163*). Sanger sequencing confirmed the mutation in the child and the wild-type sequence of the corresponding region in both unaffected parents. In patient 2, no pathogenic change in the annotated and validated *FBRSL1* transcript (NM_001142641.2) was initially detected. Since the child's clinical presentation was almost identical to that of patient 1, we manually inspected putative intronic regions of the *FBRSL1* gene, for which partial reads could be generated by Agilent's SureSelectXT Human All Exon V5 Enrichment method. For the region 12:133,085,800–133,085,880 (GRCh37/hg19), two reads were obtained in patient 2, each showing a 23-bp deletion (12:133,085,843–133,085,866; GRCh37/hg19), while in the parents only one read without deletion was observed (see Suppl. Figure 1A). To exclude the 23-bp deletion as an artifact of a poorly covered region, we used Sanger sequencing on genomic DNA of patient 2 and her parents. This confirmed a heterozygous state of the 23-bp deletion in the affected child and a wild-type status of this gene region in the healthy parents (Suppl. Figure 1B, C).

To date, 15 hypothetical transcripts are listed for human *FBRSL1* in NCBI. For *AUTS2*, the *FBRSL1* paralogue, a long transcript and shorter N- and C-terminal transcripts were validated. Therefore, it is conceivable that for *FBRSL1* also shorter N- and C-terminal transcripts exist like it is already known for the murine fbrsl1. The de novo 23-bp deletion would affect exon 3 of a hypothetical alternative N-terminal transcript (XM_005266181.4), leading to a frameshift and premature stop codon (c.581_603del).

A third patient with a truncating *FBRSL1* mutation (c.332 G > A; p.Trp111*; 12:133,085,787; GRCh37/hg19) was identified by single exome sequencing and reverse phenotyping. The mutation was not detected in the healthy mother of patient 3. The father was not available for genetic testing.

Neither of the three identified variants is listed in the gnomAD database or *Exome Aggregation Consortium* (ExAC) (Lek et al. 2016). RNA- analysis revealed in all three children in comparison to the healthy parents in case 1 and 2 and the healthy mother in case 3 that the truncating variants escaped the mechanism of nonsense mediated mRNA decay (NMD) (Suppl. Figure 2).

### FBRSL1 consists of different isoforms and all three detected mutations affect short N-terminal isoforms

**Fig. 2:**
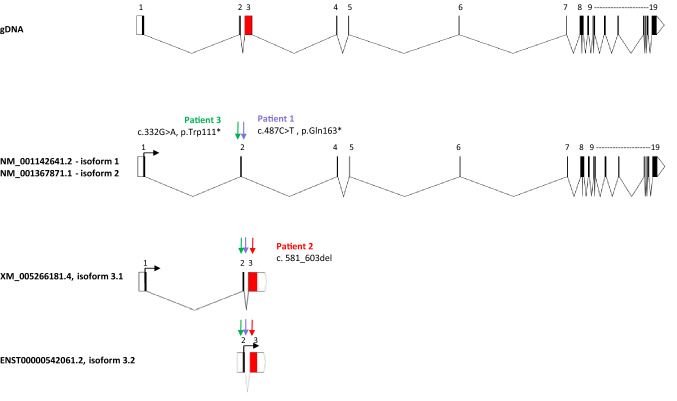
Scheme of validated human *FBRSL1* isoforms and localization of the detected mutations. The scheme was created using the Exon–Intron-Graphic Maker available at https://wormweb.org/exonintron by Nikhil Bhatla (2012) (version 4)

As the *FBRSL1* transcript variants have only insufficiently been described, we subsequently performed RT-PCR analyses on cDNA isolated from control lymphocytes and Western blot analysis using protein isolated from HEK293 cells to identify and validate protein-encoding isoforms. By RT-PCR we confirmed the existence of the hypothetical transcript XM_005266181.4 (NCBI). Compared to the validated long variant (NM_001142641.2, isoform 1, NCBI), this shorter isoform contains exons 1 and 2 and included an alternative exon 3 with a stop codon (isoform 3.1). In addition to this version 3.1, we also observed a variant consisting only of exon 2 and the alternative exon 3, using an in-frame ATG in exon 2 (isoform 3.2). This variant differs in the 5´UTR region and was annotated as a hypothetical transcript (ENST00000542061.2, ENSEMBL) (Fig. 2).

For validation of the detected 23-bp deletion on transcript level, we performed RT-PCR analysis on cDNA isolated from lymphocytes from patient 2 and her parents. This confirmed the presence of the heterozygous 23-bp deletion in patient 2 (Suppl. Figure 1B and C). Thus, the detected 23-bp deletion (12:133,085,843–133,085,866; GRCh37/hg19) affects exon 3 and can be described as c.581_603del with respect to the ATG of the transcript isoform 3.1. Isoform 3.1 encodes a hypothetical protein consisting of 589 amino acids (~ 66 kDa), while isoform 3.2 has an estimated molecular weight of 55 kDa. According to in silico analysis, both isoforms contain a DNA translocase domain (FtsK domain, NCBI conserved domains database, CDD) encoded by a part of exon 3. The long isoform 1 lacks this domain and contains instead a AUTS2 (*autism susceptibility gene* 2) domain in the C-terminal part, describing a homologous region found in the FBRSL1 paralogue AUTS2.

**Fig. 3:**
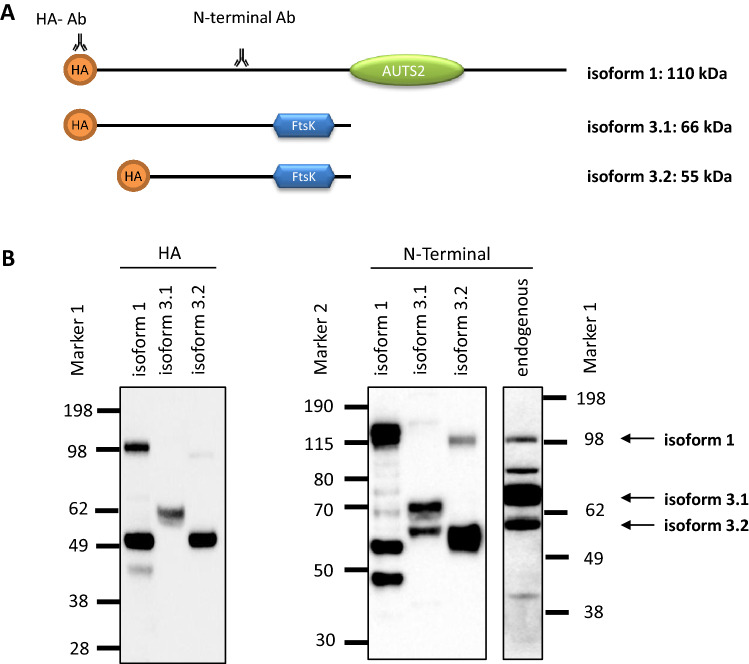
Western blot analysis of endogenous and transfected FBRSL1 isoforms. **a** Scheme representation of FBRSL1 isoforms detectable with an N-terminal antibody. The scheme was created using a domain architecture software (https://prosite.expasy.org/mydomains). Isoform 1, consisting of the AUTS2 domain, has a predicted molecular weight of 110 kDa, while isoform 3.1 has a predicted molecular weight of 66 kDa and isoform 3.2 of 55 kDa. Isoforms 3.1. and 3.2 lack the AUTS2 domain and contain a Ftsk DNA translocase domain with an unknown function. **b** Plasmids containing isoforms 1, 3.1 and 3.2 in fusion to an HA-taq were detected with either an HA-antibody (HA) or with the N-terminal FBRSL1 antibody (N-terminal) in comparison to the endogenous FBRSL1 expression of HEK293 cells. The approximately estimated sizes of 110 kDa, 66 kDa and 55 kDa of the three different isoforms were detected

Using an N-terminal antibody for Western blot analysis on protein lysates from HEK293 cells we detected an approximately 110 kDa, 66 kDa and 55 kDa band corresponding to the estimated sizes of isoforms 1, 3.1 and 3.2. Two additional bands were observed, possibly belonging to additional isoforms. Furthermore, we were able to confirm the endogenous protein bands by expression of the respected isoforms 1, 3.1 and 3.2 in fusion to an HA-taq in HEK293 cells, using the N-terminal antibody as well as an HA-antibody (Fig. 3).

The 23-bp deletion affects only the two short transcripts 3.1. and 3.2, while isoform 1 remains unaffected. The deletion leads to a frameshift with a premature stop codon at position 356 (isoform 3.1), disrupting the DNA translocase domain (amino acid position 444–511, isoform 3.1). Interestingly, the stop mutations p.Trp111* and p.Gln163* detected in the other two patients impair not only isoform 1, but also the newly validated isoforms 3.1 and 3.2. In all three cases, the DNA translocase domain is affected. We conclude that the shorter isoforms and the disruption of the DNA translocase domain are responsible for the observed recognizable phenotype.

### FBRSL1 is ubiquitously expressed and the different isoforms show different cellular localization

Expression analysis was performed by RT-PCR on human fetal and adult tissues using human cDNA panels (Clontech) as well as on RNA isolated from human lymphocytes and the two human cell lines, HeLa and HEK293. For isoform 1 we observed a ubiquitous expression pattern as described in the databases (e.g. proteinatlas.org). The same observation was made for isoform 3.2, while isoform 3.1 showed a clear expression in fetal tissues, with partial lack of expression in the adult tissues (Suppl. Figure 3).

**Fig. 4:**
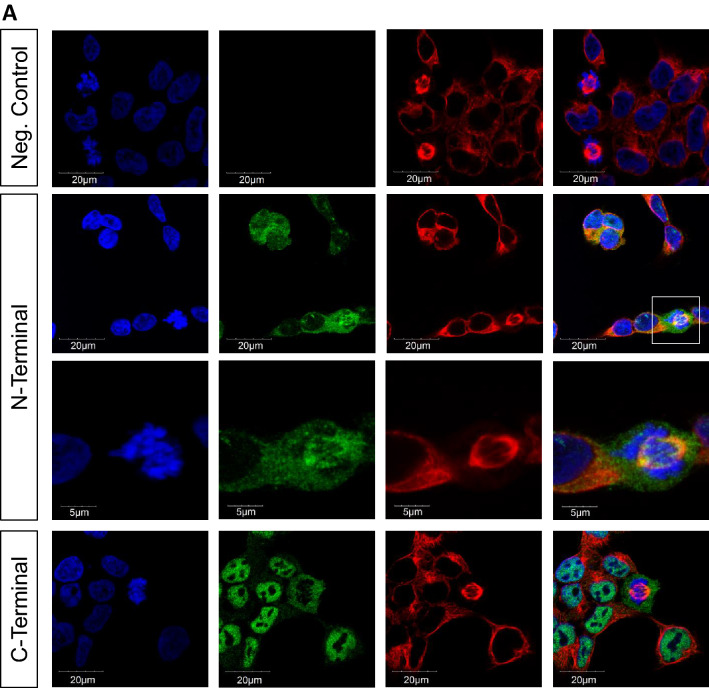

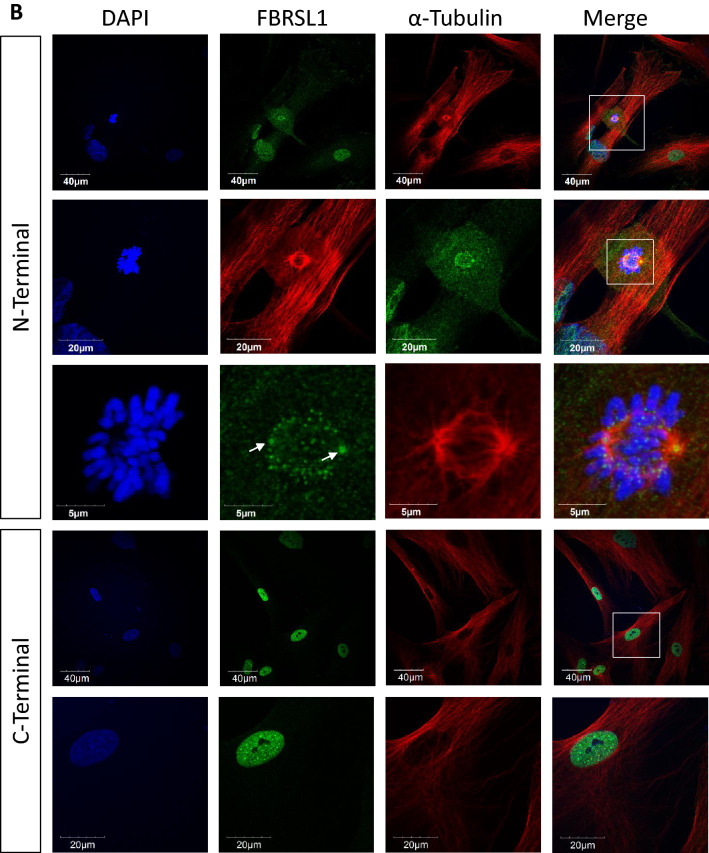
Immunofluorescence analysis performed on HEK293 cells (**a**) and human fibroblasts (**b**). The N-terminal antibody detected isoforms 1, 3.1 and 3.2 in the cytoplasm and nucleus. Interestingly, an association with centrosomes (white arrow) and kinetochores was detected. Staining with a C-terminal antibody, detecting the full-length isoform 1 and additional hypothetic short C-terminal isoforms, showed a mainly nuclear pattern without a co-localization with the mitotic spindle, centrosomes or kinetochores. α-Tubulin was used for cytoskeletal staining and nuclei were stained using DAPI. Images were obtained using a confocal laser microscope with × 600 magnification, and an additional software magnification as indicated in the respectively images

In addition, we studied the subcellular localization of FBRSL1 by immunofluorescence analysis in HEK293 cells (Fig. 4a) and human fibroblasts (Fig. 4b) by using an N-terminal and a C-terminal antibody against FBRSL1 for detection. With the C-terminal antibody, detecting full-length isoforms and potential short C-terminal isoforms, a mainly nuclear distribution was observed. With the N-terminal antibody, detecting the full-length isoform and isoforms 3.1 and 3.2, a cytoplasmic and nuclear localization was detected. In particular, N-terminal FBRSL1 isoforms appeared to be associated with the centrosomes and the kinetochores in dividing cells. We conclude therefore that FBRSL1 localizes in both nuclear and extranuclear subcellular regions depending on its isoforms.

### FBRSL1 knockdown in *Xenopus laevis* embryos results in reduction of craniofacial structures

**Fig. 5:**
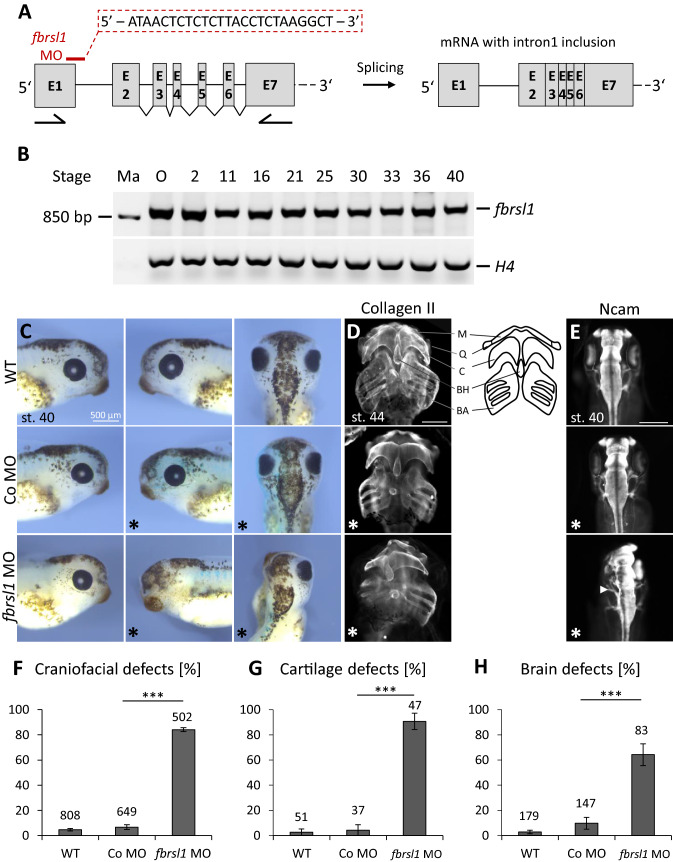
Fbrsl1 loss-of-function causes craniofacial defects in *Xenopus laevis* development. **a** Scheme of the 5′ region of *Xenopus laevis fbrsl1* with indicated exons, introns and the *fbrsl1* E1/I1 splice-blocking Morpholino target side and possible outcome (mRNA with intron 1 inclusion) after splicing. *Xenopus laevis fbrsl1* consists of 19 exons. The Morpholino sequence is given in the red dashed square. Arrows under exon 1 and exon 7 indicate the locations of the forward and reverse primer used for RT-PCR. **b** RT-PCR analysis of temporal *fbrsl1* expression in the oocyte and different developmental stages of *Xenopus* embryos. RT-PCR analysis of histone H4 serves as loading control. Ma: Marker, O: Oocyte. **c** Stage 40 wild-type and control Morpholino (10 ng) injected embryos developed normal craniofacial structures and eyes. Injection of 10 ng *fbrsl1* MO results in severe craniofacial defects and a reduction of the eye on the *fbrsl1* MO injected side (marked by *). **d** Anti-Collagen Type II immunofluorescence staining of stage 44 *Xenopus* embryos showing cartilage defects in embryos injected with *fbrsl1* MO but not in wild-type or Co MO injected embryos. M: Meckel’s, Q: quadrate, C: ceratohyal, BH: basihyal, BA: branchial arches. Scale bar represents 500 µm. **e** Anti-Ncam immunofluorescence staining of stage 40 *Xenopus* embryos shows normal brain development in control embryos, but reduced Ncam expression in embryos injected with *fbrsl1* MO. **f, g, h** The graphs summarize craniofacial, cartilage and brain defects of at least three independent experiments; number of embryos (*n*, above each bar) and standard errors of the mean are given. ***p < 0.001 in a Student’s *t*-test and a one-way ANOVA test

To explore whether the symptoms that were observed in the patients with a *FBRSL1* mutation can be phenocopied in other vertebrates, we performed Fbrsl1 loss-of-function studies in *Xenopus laevis* (Fig. 5). Analysis of the temporal *fbrsl1* expression pattern in *Xenopus laevis* revealed that *fbrsl1* is already maternally expressed in the oocyte and throughout early embryonic development from stage 2 to 40 (Fig. 5a, b). Knockdown of Fbrsl1 in *Xenopus laevis* embryos was achieved by injection of a *fbrsl1* splice-blocking morpholino (*fbrsl1* MO), blocking splicing at the exon 1/intron 1 boundary. Blocking this first splice site resulted in intron 1 inclusion (Fig. 5a), which was confirmed by RT-PCR and sequencing of the amplified cDNA fragment (Suppl. Figure 4). As intron 1 contains several in-frame stop codons, MO injection results in a severe truncation of the Fbrsl1 protein. Interestingly, *fbrsl1* MO injection caused a significant reduction of craniofacial structures and the eye in stage 40 *Xenopus laevis* embryos, whereas wild-type and control MO (co MO) injected embryos developed normally (Fig. 5c, f). As the reduction of craniofacial structures most likely results from abnormal cartilage development, we performed immunostaining of collagen II to visualize the cartilage of the head of stage 44 *Xenopus laevis* embryos. Collagen II staining revealed cartilage hypoplasia on the *fbrsl1* MO injected side of the embryos (Fig. 5d, g). The Meckel’s cartilage, the ceratohyal cartilage and the branchial arches were reduced, whereby the branchial arches were most severely affected. The basihyal cartilage, however, was not affected. As the patients carrying a FBRSL1 mutation developed microcephaly, we analyzed the development of the brain by Ncam immunostaining in *Xenopus laevis* embryos. Consistent with the symptoms of the patients, Fbrsl1 depletion in *Xenopus laevis* leads to a strong reduction of Ncam expression indicating defects in brain development at the *fbrsl1* MO injected side (Fig. 5e, h). Whole-mount in situ hybridization confirmed *fbrsl1* mRNA expression in craniofacial structures including the brain, the branchial arches and the cranial nerve at stage 31 wild-type *Xenopus* embryos (Suppl. Figure 5). The results obtained indicate that Fbrsl1 seems to function during the development of craniofacial structures in both human and *Xenopus laevis*.

### Fbrsl1 loss-of-function affects neuronal migration in *Xenopus laevis* embryos

**Fig. 6:**
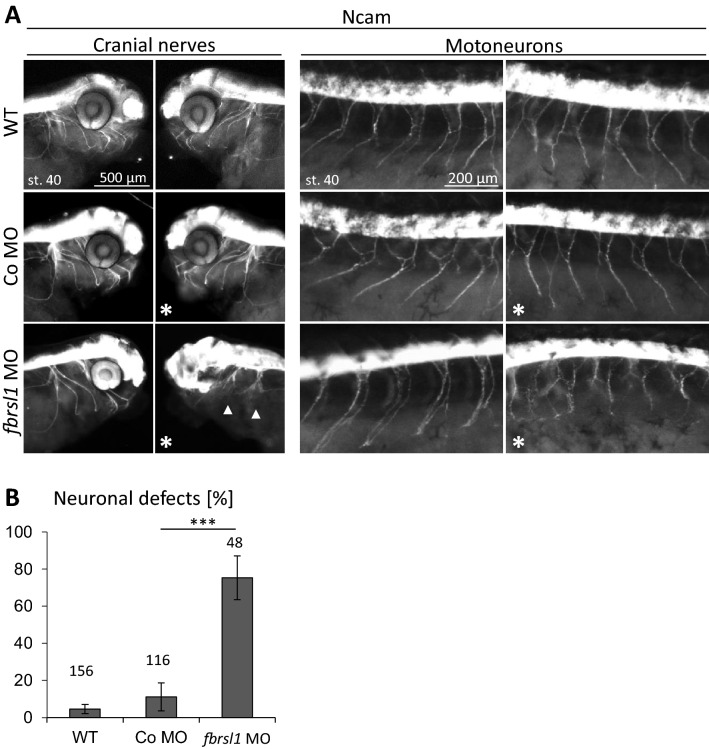
Neuronal migration is disturbed in fbrsl1 depleted embryos. **a** Anti-Ncam immunofluorescence staining of stage 40 *Xenopus laevis* embryos indicate normal neuronal migration of cranial nerves and motor neurons in wild-type and 10 ng Co MO injected embryos, but disturbed neuronal migration in embryos injected with 10 ng *fbrsl1* MO (arrow). **b** The graph summarizes three independent experiments, number of embryos (*n*, above each bar)) and standard errors of the mean are given. ***p < 0.001 in a Student’s *t*-test and a one-way ANOVA test

AUTS2 has been described to regulate neuronal migration and neuritogenesis (Hori et al. 2014; Hori and Hoshino 2017). To study whether Fbrsl1 might have similar functions, we visualized neurons using the pan-neural marker Ncam in stage 40 embryos. The cranial nerves and motor neurons on the Fbrsl1-depleted side show impaired neuronal migration, while directional neuronal migration is observed in wild-type and Co MO injected embryos. Cranial nerves grow out of the brain but fail to migrate in a directional pattern to the ventral side. Similarly, the motor neurons elongate from the spinal cord, but lose their typical chevron-shaped organization (Fig. 6a, b). Thus, it seems that Fbrsl1 might affect neuronal migration, as it has been shown for Auts2.

### The human short N-terminal FBRSL1 isoform rescues craniofacial defects caused by fbrsl1 knockdown in *Xenopus laevis* embryos

**Fig. 7:**
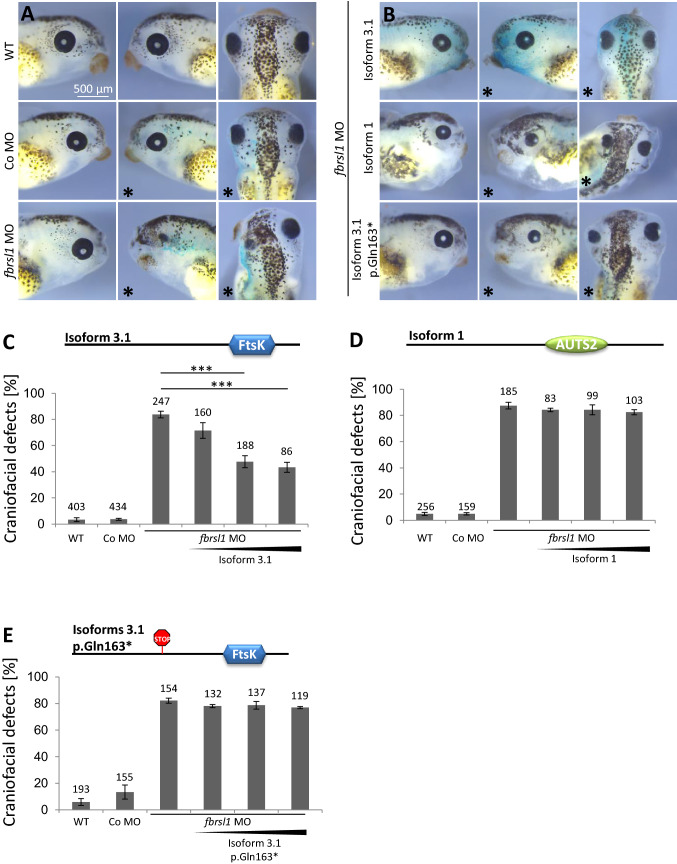
The short human N-terminal FBRSL1 isoform 3.1 can rescue craniofacial malformations induced by Fbrsl1 depletion in *Xenopus laevis.* **a** Injection of 10 ng *fbrsl1* MO caused a reduction of craniofacial structures and the eye, while wild-type and 10 ng Co MO injected embryos developed normally. **b–e** Co-injection of the human FBRSL1 isoform 3.1 significantly rescues craniofacial malformations. In contrast, co-injection of the human FBRSL1 isoform 1 or the mutated human isoform 3.1-p.Gln163* with 10 ng *fbrsl1* MO does not rescue craniofacial malformations caused by Fbrsl1 depletion. Embryos injected with 300 pg of the human FBRSL1 isoforms are shown in (**b)**. **c–e** Graphs summarizing the percentage of craniofacial defects of at least three independent experiments after co-injection of increasing concentrations (100, 200 and 300 pg) of the indicated human FBRSL1 isoforms with *fbrsl1* MO. ± s.e.m. and numbers of embryos are indicated (*n*, above each bar). Scale bar: 500 µm. ***p < 0.001 in a Student’s *t*-test and one-way ANOVA with Dunnett’s multiple comparisons test

Based on the finding that all three detected mutations in the patients affect the short N-terminal FBRSL1 isoforms 3.1 and 3.2, we hypothesized that the disruption of this shorter isoforms and thereby of the DNA translocase domain (FtsK) is crucial for the development of the clinical symptoms. Indeed, craniofacial defects caused by Fbrsl1 knockdown in *Xenopus laevis* embryos were partially rescued only by co-injection of the human FBRSL1 isoform 3.1 in a dose-dependent manner (Fig. 7a–c). Co-injection of 100 pg of the FBRSL1 isoform 3.1 led to a slight but not significant rescue of craniofacial malformations. In contrast, co-injection of 200 pg or 300 pg of FBRSL1 isoform 3.1 resulted in a significant rescue of craniofacial defects. Co-injection of the FBRSL1 isoform 1 lacking the DNA translocase domain did not rescue the craniofacial defects caused by Fbrsl1 depletion (Fig. 7b, d). Interestingly, the mutated human FBRSL1 isoform 3.1-p.Gln163* containing the p.Gln163* mutation from patient 1 also failed to rescue the craniofacial defects caused by Fbrsl1 depletion (Fig. 7a, d). These data support the hypothesis that mutations of the shorter N-terminal isoforms are causative for the described phenotype.

## Discussion

In the present study, we report three unrelated children with an hitherto undiagnosed, but recognizable malformation syndrome and a heterozygous truncating variant in *FBRSL1*. All children presented with respiratory insufficiency, feeding difficulties, postnatal growth restriction and microcephaly, global developmental delay, no active speech, contractures, facial dysmorphism and other malformations like heart defects, cleft palate and distinctive skin creases in the first year of life. The skin folds observed in two children affected preferentially the back and became less pronounced with increasing age.

In two children, we confirmed the *FBRSL1* variant to be de novo, while in the third child the father was not available for genetic testing. No other variant explaining the symptoms was detected by exome sequencing in the three affected children. The two identified nonsense mutations and the deletion in *FBRSL1* are not described in large cohorts like the *Exome Aggregation Consortium* (ExAC) or in the gnomAD database. All three mutations cluster in the N-terminal region of *FBRSL1*, affecting a hypothetical transcript isoform. We were able to demonstrate the existence of this isoform by RT-PCR and Western blot analysis in addition to another hypothetical variant, using an alternative in-frame ATG in exon 2.

*FBRSL1*, a poorly characterized gene, is a paralogue of *AUTS2*. Pathogenic mutations in *AUTS2*, mainly gross deletions, lead to a neurodevelopmental syndrome with autism, microcephaly, short stature, contractures, facial dysmorphism and, in some cases, additional malformations like heart defects (Beunders et al. 2013), demonstrating an overlap to the phenotype observed in our three patients with truncating *FBRSL1* variants. The AUTS2 syndrome phenotype can be highly variable, ranging from unaffected carriers to the full syndrome. A genotype–phenotype correlation exists. Individuals with an in-frame deletion of the N-terminal part show a milder phenotype, restricted to variable degrees of neurocognitive defects, than individuals with a deletion affecting the C-terminal part of the gene. The full AUTS2 syndrome phenotype is usually present in cases with an inactivation of the entire gene or the C-terminal part of the gene (Beunders et al. 2016).

For AUTS2, several isoforms have been described including a long isoform, counting 19 exons, a short C-terminal isoform starting from exon 9, and a short N-terminal transcript (Beunders et al. 2013; Oksenberg and Ahituv 2013; Hori and Hoshino 2017). Auts2 knockdown in zebrafish leads to morphants recapitulating part of the human phenotype including microcephaly and smaller lower-jaw size. The zebrafish phenotype was fully rescued by using the human full-length transcript and the shorter 3´ transcript (Beunders et al. 2013, 2016; Oksenberg et al. 2013). Both transcripts share a highly conserved AUTS2 domain, leading to the suggestion that the disruption of the AUTS2 domain is responsible for the phenotype.

We used the *Xenopus laevis* system for further in vivo analysis. A knockdown of Fbrsl1 leads to craniofacial abnormalities and disturbance in the outgrowth of cranial nerves and motor neurons in *Xenopus laevis* embryos. Interestingly, in contrast to the Auts2 experiments, we were able to rescue the craniofacial defects with the short N-terminal isoform, while the long isoform showed no statistically significant effect. In addition, the short N-terminal isoform bearing the mutation c.487C > T (p.Gln163*) failed to rescue the *Xenopus laevis* phenotype, confirming the pathogenicity of the mutation.

In silico analysis revealed that the used short N-terminal isoform contains a DNA translocase domain in exon 3 with unknown function. As isoform 1 lacks exon 3, this version does not contain the DNA translocase domain but contains instead in its C-terminal region the AUTS2 domain, which is missing in the short N-terminal variant. The different domain architecture of the isoforms suggests that they differ in their functional roles.

In humans, gross deletions encompassing FBRSL1 as well as neighboring genes have been reported (Decipher database). However, the phenotype of these patients, which may likely also depend on additionally affected genes, is different from our patients and for example the typical skin creases have not been reported. Moreover, isoform 1 seems to be tolerant to loss of function (LoF) mutations (pLI = 0.01; gnomAD database); indeed there are truncating alleles listed in gnomAD for this transcript version, suggesting that truncated protein variations exist in the normal, healthy population. Thus, for the long FBRSL1 transcripts (isoform 1 and 2) haploinsufficiency appears to be unlikely.

In our three patients the two, short N-terminal, FBRSL1 transcripts are affected. Although truncating variants have been described (gnomAD) for exon 1, so far no truncating variants have been observed in the newly characterized short N-terminal transcript using an in-frame ATG in exon 2 (isoform 3.2). Thus, the underlying disease mechanism could be due to haploinsuffiency of the isoform 3.2 and a resulting misbalance between the different FBRSL1 isoforms.

Nevertheless, as truncating alleles of all three patients escaped nonsense-mediated mRNA decay (NMD) a dominant-negative mechanism for the detected variants is also conceivable. For example, for microtubule associated proteins like α-tubulin a dominant-negative effect was demonstrated for some variants (Aiken et al. 2019). It was suggested that multiple tubulin isotypes can partially compensate for a heterozygous deletion of a tubulin gene, but may not overcome an altered microtubule function due to dominant-negatively acting variants. The same mechanism could be possible for a gene like human FBRSL1, which has different isoforms and paralogues. Further studies are needed to clarify this aspect.

The fact that the isoforms have a different subcellular localization supports the hypothesis of different functional roles. With the C-terminal antibody, detecting full-length and possible short N-terminal isoforms, a nuclear pattern was observed, while with the N-terminal antibody, detecting the full-length isoform and isoforms 3.1 and 3.2, a predominant cytoplasmatic localization was detected. Furthermore, the short N-terminal FBRSL1 isoforms show a co-localization with the centrosomes and the kinetochores, suggesting a role in microtubule-kinetochore organization, as well as a possible function in correct cell division. Centrosomes, the microtubule-organizing centers of the cells, are described to play an important role during embryonic CNS growth and neurogenesis by controlling appropriate cell divisions. The disturbance of this mechanism is suggested to be responsible for neurodevelopmental disorders and microcephaly (Saade et al. 2018), a symptom seen in all three patients.

Microtubules are known to be involved in different mechanisms like proper neural crest cell migration and outgrowth of neurites, nuclear translocation, chromatid separation and intracellular trafficking (Breuss and Keays 2014; Breuss et al. 2017). Skin development and stratification occur through asymmetric cell divisions in which the orientation of the mitotic spindle plays an important role (Lechler and Fuchs 2005; Williams et al. 2011). Regarding the circumferential skin folds observed in CSCSC syndrome patients, it was suggested that the underlying cause are defects in cell division leading to an altered progenitor output (Isrie et al. 2015). A disturbed microtubule-kinetochore function could explain main parts of the observed symptoms in our patients via defects in neural crest cell migration and neurite outgrowth and cell division problems.

We conclude that the disruption of the N-terminal isoforms, affected in all three patients, at critical points in embryogenesis lead to a neurodevelopmental syndrome with similarities to the CSCSC syndrome and the AUTS2 syndrome.

## Web resources

DECIPHER, https://decipher.sanger.ac.uk/

ExAC Browser, https://exac.broadinstitute.org/ now included into https://gnomad.broadinstitute.org/

gnomAD Browser, https://gnomad.broadinstitute.org/

## Electronic supplementary material

Below is the link to the electronic supplementary material.

Supplementary file1 Suppl. Figure 1: Exome data analysis with Varbank (https://varbank.ccg.uni-koeln.de) and results of Sanger sequencing of patient 2 as well as her parents and results of RT-PCR analysis A. For patient 2, only two reads were observed in Varbank for region 12:133085800-133085880 (GRCh37/hg19), each of which showed a 23-bp deletion (12:133085843-033085866), while for the parents only one read without deletion was detected. Sanger sequencing of genomic DNA for this region confirmed the wild-type sequence in the healthy parents and indicated a heterozygous status (frameshift) for the 23-bp deletion in the patient. B. Gel electrophoresis of the RT-PCR analysis on RNA isolated from lymphocytes of the affected child 2 and her parents. H_2_O was used as negative control. Gel extraction was used to sequence the detected bands, solely. Three bands were detected in the affected child, while only one band, correlating to the size of the wild-type band, was detected in the parents. C. After Sanger sequencing, the upper band (one star) was identified as a duplex from a wild-type product and a deleted product. The middle band (2 stars) corresponds to the wild-type sequence. The lower band (3 stars) contains the 23-bp deletion. (PDF 454 kb)

Supplementary file2 Suppl. Figure 2: RNA-Analysis revealed that all three muations escaped the mechanism of NMD. Reads mapped to gene *FBRSL1* were visualised using Integrated Genome Viewer (IGV) version 2.8.2, with the samples ordered by family (patient1 with parents 1, patient 2 with parents 2 and patient 3 with mother 3). The reads were scaled by family, such that the read counts of *FBRSL1* for each child are directly comparable to the read counts of *FBRSL1* in the parents. The annotation (bottom panel) displays two tracks, the first being of *FBRSL1* annotated by ENSEMBL hg38 version 97, and the second containing custom annotation of *FBRSL1* including exon 3 (PDF 283 kb)

Supplementary file3 Suppl. Figure 3: Expression analysis performed by RT-PCR on human fetal and adult tissues using cDNA panels (Clontech). In addition RT-PCR analysis was performed on RNA isolated from human lymphocytes and HeLa and HEK293 cells. For isoform 1, a ubiquitous expression pattern was observed, as well as for isoform 3.2, while isoform 3.1 shows a clear expression in fetal tissues, with partial lack of expression in the adult tissues (PDF 234 kb)

Supplementary file4 Suppl. Figure 4: *Fbrsl1* MO blocks the exon 1/intron 1 splice site of *fbrsl1*. A Scheme of a part of the *Xenopus laevis fbrsl1* with the *fbrsl1* splice-blocking Morpholino binding site. The *fbrsl1* splice-blocking Morpholino targets the exon 1/intron 1 splice site. The locations of the forward and reverse primer are indicated. B RT-PCR using the indicated primer pair results in the amplification of a ~ 900 bp band from cDNA isolated from 10 ng *fbrsl1* MO injected embryos but not from cDNA isolated from 10 ng Co MO injected embryos. C Sequence alignment of the amplified band confirmed inclusion of intron 1. Exon 1 (marked in yellow) and a part of intron 1 were detected. Red stars * mark the location of in-frame stop codons. Similar results were obtained from three independent experiments. (PDF 535 kb)

Supplementary file5 Suppl. Figure 5: Whole-mount in situ hybridization of *fbrsl1* mRNA in stage 31 wild-type Xenopus embryos. *Fbrsl1* is expressed in the head of tailbud Xenopus embryos. br: brain, cn: cranial nerves, ba: branchial arches. (PPTX 10390 kb)

## Data Availability

The raw whole-exome sequencing data of the affected children and their parents cannot be made publicly available for reasons of individual’s confidentiality. All other in this study generated data are included in the article (and its supplemental data).
