# Supplementary material for: De novo mutations in FBRSL1 cause a novel recognizable malformation and intellectual disability syndrome
Source: Hum Genet. 2020 May 18;139(11):1363–79. doi: 10.1007/s00439-020-02175-x (PMC7519918; doi:10.1007/s00439-020-02175-x)

Suppl. Figure 1

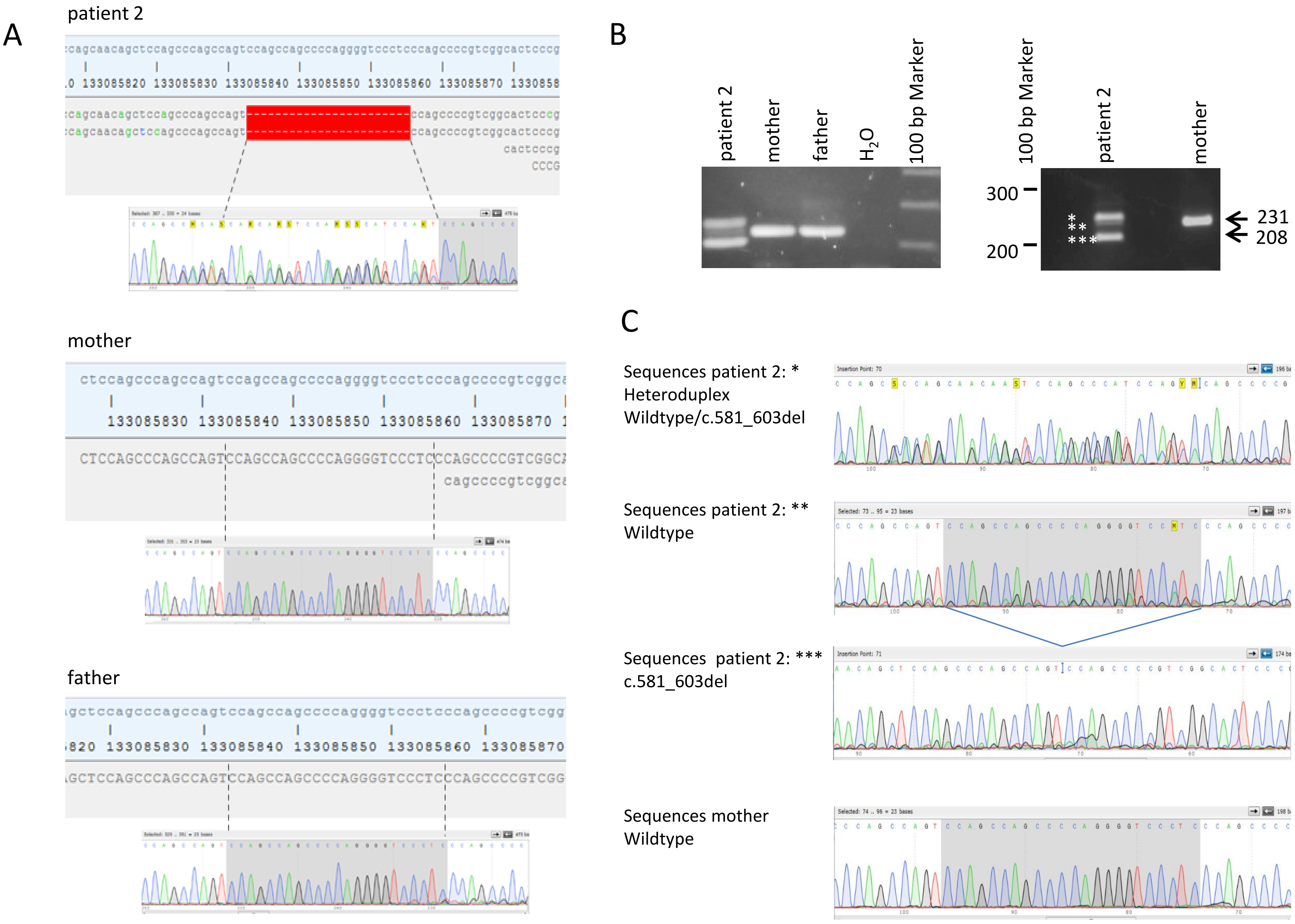

Supplement: Supplementary file 1 — Supplementary file1 Suppl. Figure 1: Exome data analysis with Varbank (https://varbank.ccg.uni-koeln.de) and results of Sanger sequencing of patient 2 as well as her parents and results of RT-PCR analysis A. For patient 2, only two reads were observed in Varbank for region 12:133085800-133085880 (GRCh37/hg19), each of which showed a 23-bp deletion (12:133085843-033085866), while for the parents only one read without deletion was detected. Sanger sequencing of genomic DNA for this region confirmed the wild-type sequence in the healthy parents and indicated a heterozygous status (frameshift) for the 23-bp deletion in the patient. B. Gel electrophoresis of the RT-PCR analysis on RNA isolated from lymphocytes of the affected child 2 and her parents. H2O was used as negative control. Gel extraction was used to sequence the detected bands, solely. Three bands were detected in the affected child, while only one band, correlating to the size of the wild-type band, was detected in the parents. C. After Sanger sequencing, the upper band (one star) was identified as a duplex from a wild-type product and a deleted product. The middle band (2 stars) corresponds to the wild-type sequence. The lower band (3 stars) contains the 23-bp deletion. (PDF 454 kb) [file 439_2020_2175_MOESM1_ESM.pdf]
