# Supplementary figures and images for: De novo mutations in FBRSL1 cause a novel recognizable malformation and intellectual disability syndrome
Source: Hum Genet. 2020 May 18;139(11):1363–79. doi: 10.1007/s00439-020-02175-x (PMC7519918; doi:10.1007/s00439-020-02175-x)

## Suppl. Figure 2

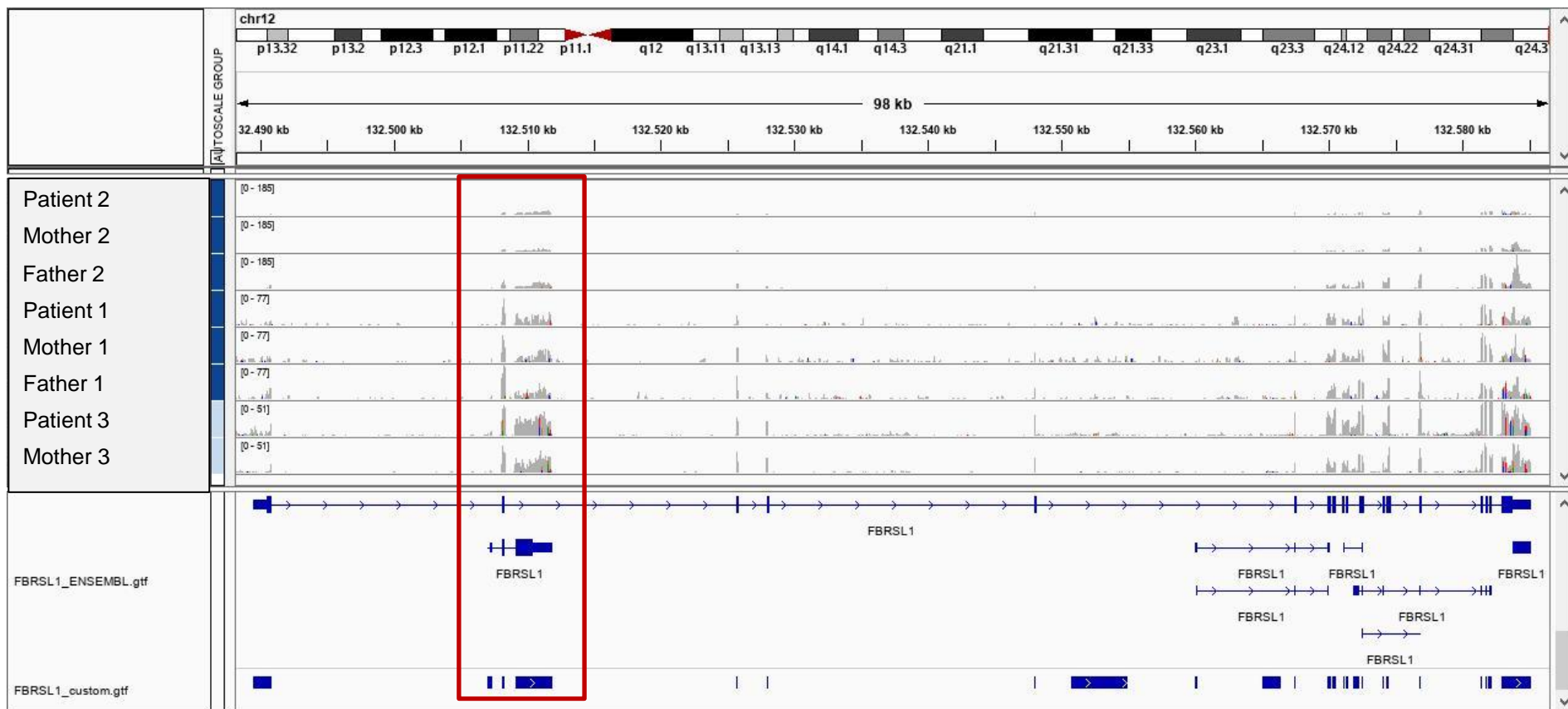

Supplement: Supplementary file 2 — Supplementary file2 Suppl. Figure 2: RNA-Analysis revealed that all three muations escaped the mechanism of NMD. Reads mapped to gene FBRSL1 were visualised using Integrated Genome Viewer (IGV) version 2.8.2, with the samples ordered by family (patient1 with parents 1, patient 2 with parents 2 and patient 3 with mother 3). The reads were scaled by family, such that the read counts of FBRSL1 for each child are directly comparable to the read counts of FBRSL1 in the parents. The annotation (bottom panel) displays two tracks, the first being of FBRSL1 annotated by ENSEMBL hg38 version 97, and the second containing custom annotation of FBRSL1 including exon 3 (PDF 283 kb) [file 439_2020_2175_MOESM2_ESM.pdf]

### Suppl. Figure 3

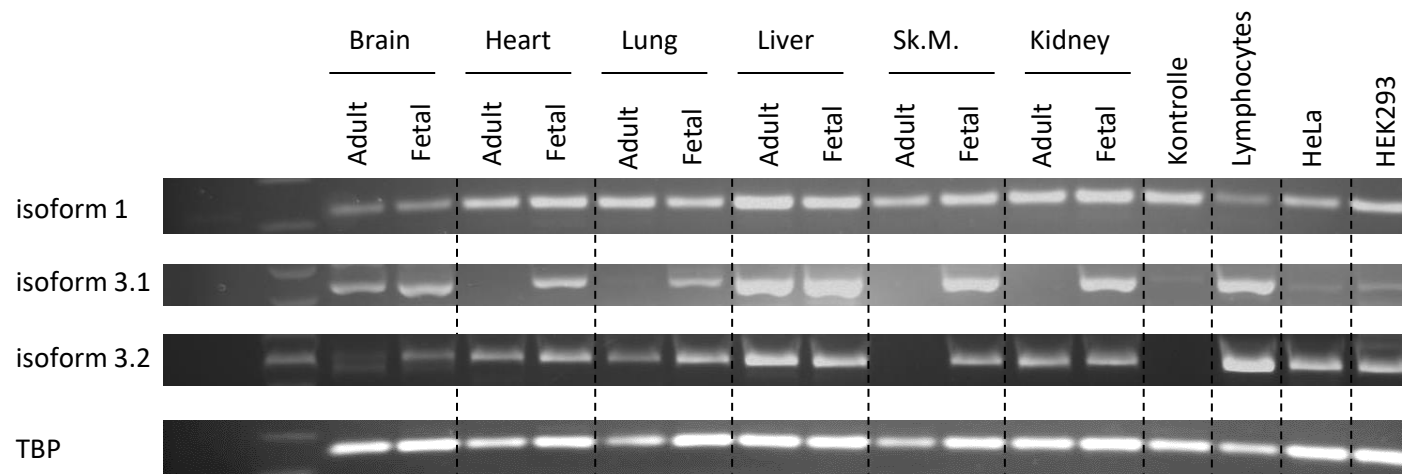

Supplement: Supplementary file 3 — Supplementary file3 Suppl. Figure 3: Expression analysis performed by RT-PCR on human fetal and adult tissues using cDNA panels (Clontech). In addition RT-PCR analysis was performed on RNA isolated from human lymphocytes and HeLa and HEK293 cells. For isoform 1, a ubiquitous expression pattern was observed, as well as for isoform 3.2, while isoform 3.1 shows a clear expression in fetal tissues, with partial lack of expression in the adult tissues (PDF 234 kb) [file 439_2020_2175_MOESM3_ESM.pdf]

## Suppl. Figure 4

**A**

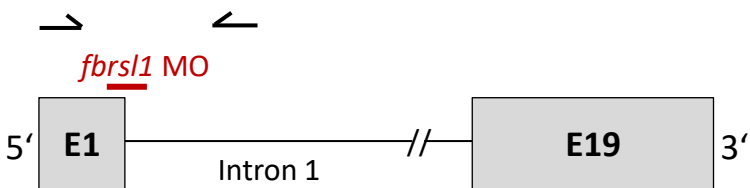

**B**

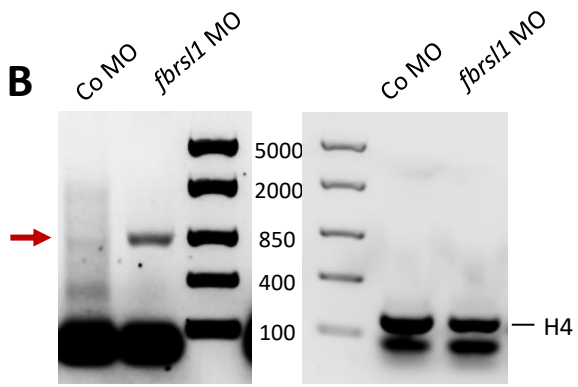

**C**

[illegible]

Supplement: Supplementary file 4 — Supplementary file4 Suppl. Figure 4: Fbrsl1 MO blocks the exon 1/intron 1 splice site of fbrsl1. A Scheme of a part of the Xenopus laevis fbrsl1 with the fbrsl1 splice-blocking Morpholino binding site. The fbrsl1 splice-blocking Morpholino targets the exon 1/intron 1 splice site. The locations of the forward and reverse primer are indicated. B RT-PCR using the indicated primer pair results in the amplification of a ~ 900 bp band from cDNA isolated from 10 ng fbrsl1 MO injected embryos but not from cDNA isolated from 10 ng Co MO injected embryos. C Sequence alignment of the amplified band confirmed inclusion of intron 1. Exon 1 (marked in yellow) and a part of intron 1 were detected. Red stars * mark the location of in-frame stop codons. Similar results were obtained from three independent experiments. (PDF 535 kb) [file 439_2020_2175_MOESM4_ESM.pdf]

## Slide 1
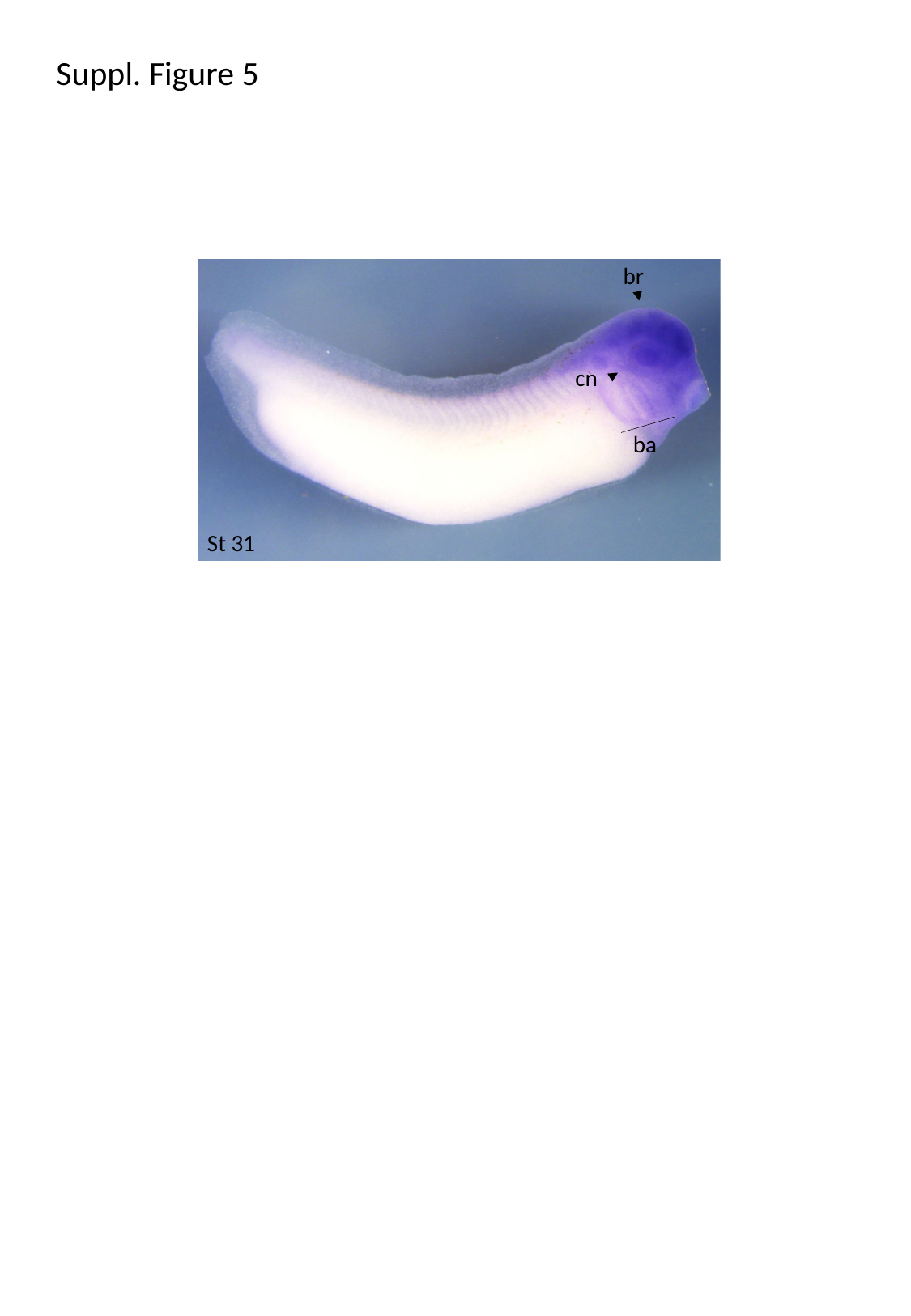

Suppl. Figure 5
br
cn
ba
St 31

Supplement: Supplementary file 5 — Supplementary file5 Suppl. Figure 5: Whole-mount in situ hybridization of fbrsl1 mRNA in stage 31 wild-type Xenopus embryos. Fbrsl1 is expressed in the head of tailbud Xenopus embryos. br: brain, cn: cranial nerves, ba: branchial arches. (PPTX 10390 kb) [file 439_2020_2175_MOESM5_ESM.pptx]
